# Characterisation of the indigenous knowledge used for gastrointestinal nematode control in smallholder farming areas of KwaZulu-Natal Province, South Africa

**DOI:** 10.1186/s12917-022-03172-0

**Published:** 2022-02-21

**Authors:** Sithembile Z. Ndlela, Mbusiseni V. Mkwanazi, Michael Chimonyo

**Affiliations:** 1 Animal and Poultry Science, School of Agricultural, Earth and Environmental Sciences, University of KwaZulu-Natal, P Bag X01 Scottsville 3209, Pietermaritzburg, South Africa; 2 Faculty of Science, Engineering and Agriculture, University of Venda, Limpopo Province, P Bag X5050, Thohoyandou, 0950 South Africa

**Keywords:** Anthelmintic plants, Ethnoveterinary knowledge, Helminthiasis, Roundworms, Small ruminants

## Abstract

**Background:**

The use of indigenous knowledge (IK) to control gastrointestinal nematodes has been known since ancient times. The objective of the study was to characterise the use of indigenous knowledge to control gastrointestinal nematodes in goats.

**Methods:**

A structured questionnaire was used to collect data from farmers. Chi-square was used to compute associations; the generalized linear model was used for mean rank scores.

**Results:**

Roundworms were the most common gastrointestinal nematode (GIN) affecting goats reared in the bushland vegetation type than grasslands. Twelve plant species were commonly used to control GIN in goats, with *Cissus quadrangularis* Linn. singled out as the most widely used plant with a use-value of 0.97, followed by *Albizia anthelminthica* Brongn. (0.66), *Cissus rotundifolia* (Forssk.) Vahl (0.63), *Vachellia xanthophloea* (Benth.) P.J.H. Hurter (0.59), *Aloe marlothii* A. Berger (0.58), *Sclerocarya birrea* (A. Rich.) Hochst (0.54), *Gomphocarpus physocarpus* E. Mey (0.53), *Aloe maculata* All. (0.50), *Trichilia emetica* Vahl (0.47), *Aloe ferox* Mill. (0.43), *Vernonia neocorymbosa* Hilliard (0.25) *and Schkuhria pinnata* (Lam) Kuntze ex Thell (0.16). *C. rotundifolia*, *V. xanthophloea*, *S. birrea* and *T. emetica* were dominant plant species used to control GIN in goats reared in the grassland vegetation. *A. maculata*, *A. ferox* and *V. neocorymbosa* were dominant in the bushland vegetation type.

**Conclusion:**

The study revealed that ethnoveterinary plants are widely used in grassland and bushland vegetation types to control GIN in goats. Scientific validation of their efficacy and safety should be carried out to provide a cheaper alternative, thus improving the community livelihoods and development.

**Supplementary Information:**

The online version contains supplementary material available at 10.1186/s12917-022-03172-0.

## Introduction

Goats contribute to economic, religious and socio-cultural enrichment and symbolize prestige in resource-limited areas [1]. Goats remain predominant due to their low input requirements and ability to adapt to harsh environmental conditions prevalent in these areas [2] In addition, they have comparative advantages over livestock species such as cattle and sheep due to their efficient use of available feeding resources and rapid turnover [3] The increasing human population size reduces the grazing land for cattle and exacerbates the lack of fodder, thus creating room for goats to take precedence [4] Although goats possess such worthy attributes, however, their productivity in resource-limited areas is constrained. The prevalence of the long dry season coupled with drought has a negative impact on goat productivity. It causes variation in the quality and quantity of vegetation, affecting the nutrition and immunity of goats and the life cycle of the parasitic helminth [5]. Gastrointestinal parasitic infections are a worldwide challenge with greater impact in the Sub-Saharan region due to warm temperatures, poor management practices, and inadequate control measures [6–8].

Gastrointestinal nematodes are usually controlled using anthelmintic drugs; however, their efficiency has decreased over the years due to various factors such as incorrect dosages, repeated use and use of low-quality drugs [9]. Such incongruities render anthelmintics unsustainable due to the development of parasite resistance, which is widespread worldwide and threatens their utilisation [10]. The resistance of parasites to anthelmintic drugs, unsustainable provision of drugs by government institutions, inability to reach medication shops, extortionate prices of drugs, and chemical residue in animal products limit the use of anthelmintics [11].

Efforts to develop sustainable integrated novel approaches that are non-chemical to treat GIN, such as the use of indigenous knowledge (IK) are, required. Indigenous knowledge is the local cumulative and dynamic body of knowledge and skills unique to native people developed from centuries of interaction with the natural environment [4]. Indigenous knowledge is part of a community-based approach that has been providing basic services, such as veterinary care, to resource-limited farmers in the past decades. To date, approximately 80% of the world population predominantly relies on IK for the welfare of their livestock, including goats [12]. For example, when goats are infested with gastrointestinal parasites, plants such as *Agapanthus praecox* are used to control parasites [13]. Plants produce a wide range of secondary metabolites that play several roles, such as controlling diseases and parasites, which possess chemical structures that are not present in synthetic compounds [14].

Indigenous knowledge is passed from generation to generation orally and there is a danger that it may be altered or lost due to acculturation, technical and socio-economic changes [13]. Indigenous knowledge plays a vital role in grassroot development to empower communities by enhancing their knowledge and resources for sustainable development and should thus, be encouraged and promoted. Sharing IK within and across communities could help enhance cross-cultural understanding and promote the cultural dimension of development. Understanding the utilization of IK provides a scope to design activities to help communities and strengthen the contribution of IK to livestock veterinary care. The objective of the study was to characterise the use of indigenous knowledge to control gastrointestinal nematodes by goat farmers of the KwaZulu-Natal Province in South Africa. This area is the goat farming zone in South Africa, where indigenous knowledge is widely practiced. The indigenous knowledge will benefit national and international communities in the fight against sustainable development challenges and in maintaining global biodiversity. It will contribute to the sustainability and productivity of the ecosystems.

## Materials and methods

### Description of the study site

The study was conducted at Jozini municipality of Umkhanyakude district in the Northern part of KwaZulu-Natal Province, South Africa. The study site is described by Ndlela et al. [15].

The location map of the study area is shown in Fig. 1 of Ndlela et al. [15].

**Fig. 1:**
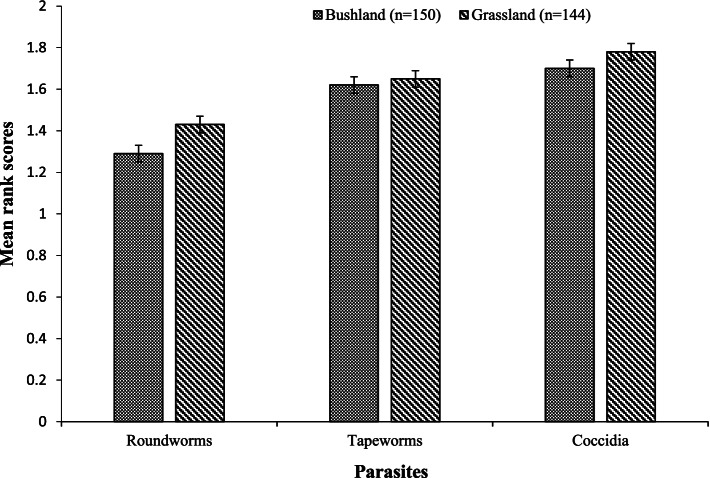
Common gastrointestinal parasites perceived by farmers to infect goats in the study site (lower mean rank score indicates greater importance)

The study was conducted in the following randomly selected villages that are amongst communities active in goat production. Communities were grouped according to the vegetation type, where Nyawushane, Mkhonjeni, Madonela, Makhonyeni have the grassland type, while Biva, Mamfene, Mkhayana, Gedleza have the bushland type. The grassland type is characterized by the dominance of wooded grasslands, where lands are covered by grasses and other herbs with woody plants. In contrast, bushland is dominated by diverse species of trees. The grassland type has a variety of plant species likely to possess anthelmintic properties.

### Data collection

A pre-assessment was conducted on 39 farmers through face-to-face interviews, and results were used to formulate a questionnaire. Structured questionnaires (*n* = 294) were administered in the local vernacular IsiZulu by trained enumerators obtained from local communities. Meetings with local authorities, livestock officers, veterinarians, farmer’s association, and extension officers were conducted according to Ndlela et al. [15]. Households were selected based on goats’ ownership, the use of IK, and willingness to participate in the study.

Data were collected on household demographics, the socio-economic status of households, livestock species kept by farmers, and parasite species frequency in goats and how farmers identify infective larvae and adult worms using IK. The questionnaire also included questions on methods used to control GIN, sources of IK, and IK used to control GIN. Plants were identified and collected in the field with assistance from IK holders. Plant specimens were authenticated at the Bews Herbarium, University of KwaZulu-Natal.

### Statistical analyses

Data were analysed using SAS [16]. The PROC FREQ procedure for chi-square was used to compute associations between household demographics, livestock herd sizes and indigenous knowledge use. A Generalized Linear Model was used to rank [2] parasite species frequency, and common gastrointestinal parasitesin the study area. The use-value of plant species is a quantitative method that indicates the relative importance of locally known plant species. The UV closer to 1 indicates more user reports for a particular plant and its importance among participants [17]. It was calculated using this formula: UV = U/N, where UV—is a use-value of the species, U – is the number of citations per species, N – is the number of informants.

## Results

### Household demographics of respondents and use of indigenous knowledge

The association between household characteristics and socio-economic status of farmers using IK are shown in Table 1. There was an association (*P* < 0.05) between the IK use and gender, where males used more IK in the bushland vegetation type than the grasslands. An association (*P* < 0.05) between IK use and the educational status of farmers was observed, where informally educated farmers used more IK in both vegetation types than formally educated farmers. Farmers with a traditional belief used more IK than Christians in both vegetation types. Unemployed farmers used more IK than employed farmers in both vegetation types.

**Table 1:** Socio-economic characteristics of respondents from Jozini and association with the indigenous knowledge

Characteristics	Bushland (%) (*n* = 150)	Grassland (%) (*n* = 144)	*x*^2^ value	Significance
**Goats’ ownership**				
Male	59	49	2.99	*
Female	41	51		
**Age distribution**				
18–30	4	6		
31–50	38	43	0.66	NS
> 50	58	51		
**Educational status**				
Formal	35.7	39.9	0.060	*
Informal	64.3	60.1		
**Religious belief**				
Traditional	49.1	39.0		
Christianity	28.1	30.8	0.029	*
Both	22.8	30.2		
**Household income per month**				
0—R1000	31.1	43.1		
R1000 -R3500	40.2	42	0.511	NS
> R3500	28.7	25.9		
**Employment status**				
Unemployed	95.4	94.4	0.312	*
Employed	5.2	5.7		

Both – represents believing in the tradition and Christianity *P < 0.05, **P < 0.01, NS – not significant *P* > 0.05

*x*^2^ – represents a Chi-square value

### Livestock species kept by farmers

Most households owned different livestock species, mainly goats (Table 2), cattle, sheep, pigs, and chickens. There was an association between IK use and livestock ownership in goats, cattle and chickens (P < 0.05). Indigenous knowledge was mainly used at herd sizes of < 20 in goats, < 28 in cattle,, and < 32 in chickens. The less use of IK is presented at large herd sizes of > 70 in goats, > 56 in cattle,, and > 80 in chickens.

**Table 2:** The proportion of goat herd sizes of farmers that are using indigenous knowledge (%)

Livestock species	IK use	*x*^2^	Significance
Goats			
< 20	70.00	8.79	^*^
21–30	16.67		
31–40	8.33		
41–50	1.18		
51–60	0.83		
61–70	0.72		
> 70	0.70		

^*^P < 0.05, *NS* Not significant

### Common goat parasites identified by participants

Gastrointestinal parasites were identified as the most important parasites affecting goat productivity. Roundworms were ranked as the most important gastrointestinal parasites with a higher importance in the bushlands than the grasslands, followed by tapeworms and coccidia (Fig. 1).

### Methods that farmers used to control gastrointestinal parasites

**Fig. 2:**
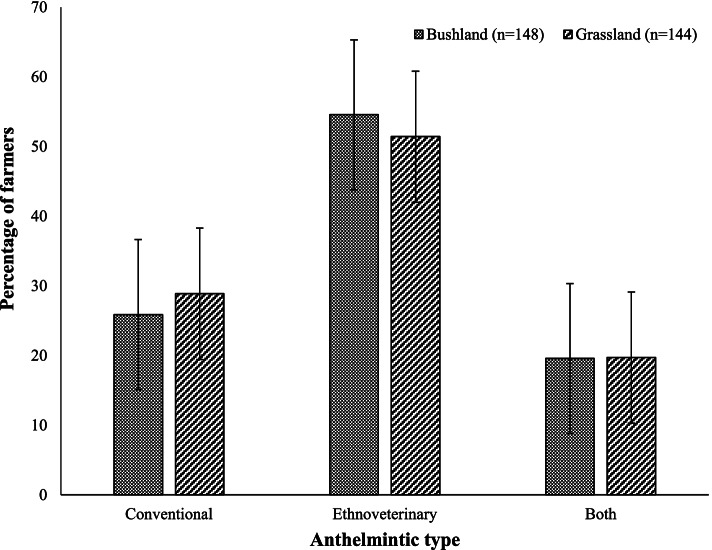
Methods used to control gastrointestinal nematodes

Figure 2 shows different methods that farmers use to control gastrointestinal parasites. Over 50% of farmers that rear goats in the bushland and grassland vegetations use ethnoveterinary medicines only to control GIN. The use of conventional drugs only was lower than ethnoveterinary medicine, but the same in both environments. Other farmers (19%) used both conventional and ethnoveterinary medicine to control GIN in goats reared in the bushlands and grasslands.

### Sources of indigenous knowledge used to control gastrointestinal nematodes

**Fig. 3:**
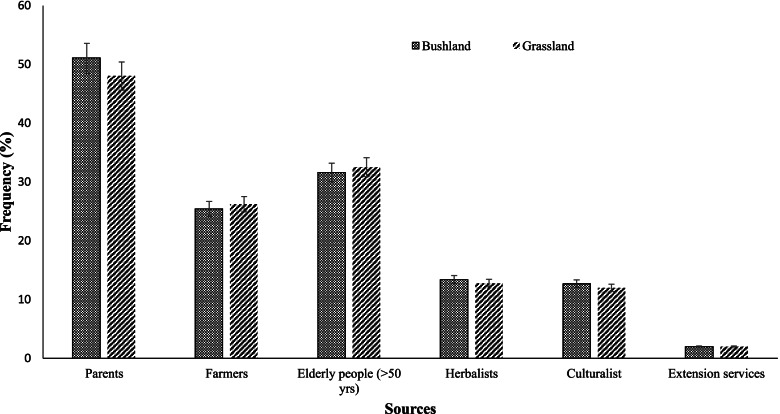
Sources of indigenous knowledge used to control gastrointestinal parasites in the study area

Sources of indigenous knowledge used to control gastrointestinal parasites in goats are shown in Fig. 3. Farmers indicated that family members (51%) are the main source of IK, followed by 32% of elderly people in the community (older than 50 years) and other farmers (25%). Herbalists and culturalists were other IK sources in the area (13% of each, respectively). Extension services were ranked as the least important reason for using IK to control GIN.

### Indigenous knowledge used by participants to control gastrointestinal nematodes in goats

The most common indigenous plants, part of the plant used, voucher numbers, methods of preparation and dosages are summarised in Table 3. The most popular plants by use-value were *Cissus quadrangularis* Linn. (0.97), *Albizia anthelminthica* Brongn. 0.66) and *Cissus rotundifolia* (Forssk.) Vahl (0.63) (Table 4). Other plant species reported were *Vachellia xanthophloea* (Benth.) P.J.H. Hurter, *Aloe marlothii* A. Berger*, Sclerocarya birrea* (A. Rich.) Hochst, *Gomphocarpus physocarpus* E. Mey, *Aloe maculata* All., *Trichilia emetica* Vahl and *Aloe ferox* Mill. (0.43–0.59) of use. *Vernonia neocorymbosa* Hilliard and *Schkuhria pinnata* (Lam) Kuntze ex Thell were 0.25 and 0.16, respectively*.* Leaves were identified as the most used part of the plant, followed by barks.

**Table 3:** Common indigenous plants used to control gastrointestinal nematodes in goats

Plant name	Family	Vernacular name	Plant part	Voucher no	Preparation method	Dosage
*A. anthelmintica*	Fabaceae	Umnala	Bark/roots	NU0068151	Decoction. The bark could also be dried, ground & mixed with feed	1 mug = adult goat, ½ mug = kid
*A. ferox*	Asphodelaceae	Inkalane	Leaves	NU0068138	Infusion	500 ml = adult goat, 250 ml = kid
*A. maculata*	Asphodelaceae	Icena/Isithezi	Leaves	NU0068164	Infusion	1 cup = adult goat, ½ cup = kid
*A. marlothii*	Asphodelaceae	Inhlaba	Leaves	NU0068166	Infusion Aloe is dried, ground and burnt to make snuff (Isinemfu)	500 ml = adult goats, 1 cup = kid 1 spoon = adult goat, ½ spoon = kid
*C. quadrangularis*	Vitaceae	Inhlashwana	Leaves (aerial part)	NU0068142	Decoction or infusion	700 ml = adult goat, 350 ml = kid
*C. Rotundifolia*	Vitaceae	Umtshovane	Leaves	NU0068158	Infusion of leaves	700 ml = adult goat, 350 ml = kid
*G. physocarpus*	Apocynaceae	Uphehlecwathi	Leaves	NU0083347	Infusion of leaves. Leaves could be ground and mixed with milk for kids	700 ml = adult goat, 350 ml = kid
*S. pinnata*	Asteraceae	Ikhambi lesisu	Whole plant	NU0068157	Decoction	1 mug = adult goat, ½ mug = kid
*S. birrea*	Anacardiaceae	Umganu	Bark	NU0068149	Decoction	1 mug = adult goat, ½ mug = kid
*T. emetica*	Meliaceae	Umkhuhlu	Bark	NU0068135	Decoction	1 mug = adult goat, ½ mug = kid
*V. xanthophloea*	Fabaceae	Umkhanyakude	Leaves/bark	NU0068155	Leaves & bark mixed with feed	-
*V. neocorymbosa*	Asteraceae	Uhlunguhlungu	Leaves	NU0068161	Infusion of leaves. Decoction of roots	1L = adult goat, 500 ml = kid

Infusion: soaking in water at room temperature overnight; Decoction: heating in water to a boiling point

**Table 4:** Conditions controlled by the documented plant species and analysis of their use-value in the study area

Plant name	Conditions controlled	Times cited (*n* = 294)	Use-value (per species)
*C. quadrangularis*	GIN, wounds, ticks	285	0.969
*A. anthelmintica*	GIN	193	0.656
*C. Rotundifolia*	GIN	184	0.626
*V. xanthophloea*	GIN	174	0.592
*A. marlothii*	GIN, diarrhoea, anaplasmosis	169	0.575
*S. birrea*	GIN	160	0.544
*G. physocarpus*	GIN, ticks	156	0.531
*A. maculata*	GIN	148	0.503
*T. emetica*	GIN	139	0.473
*A. ferox*	GIN	125	0.425
*V. neocorymbosa*	GIN	72	0.245
*S. pinnata*	GIN	48	0.163

*GIN* Gastrointestinal nematode

**Fig. 4:**
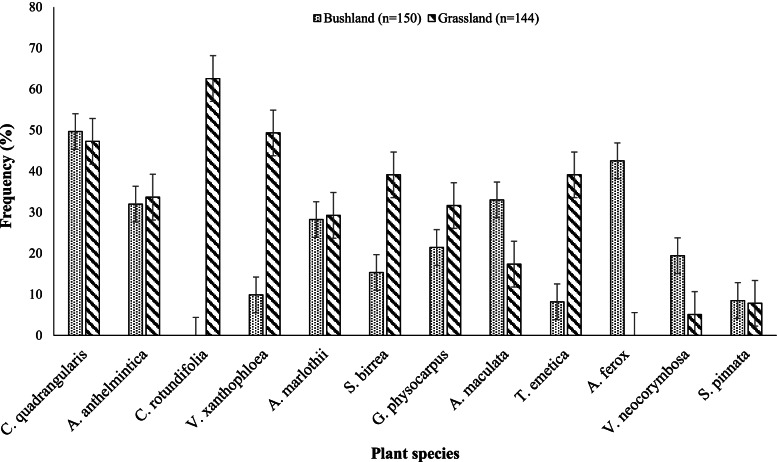
The most used anthelmintic plants by frequency

*C. rotundifolia*, *V. xanthophloea*, *S. birrea* and *T. emetica* were dominant plant species used to control GIN in goats reared in the grassland vegetation (Fig. 4). *A. maculata*, *A. ferox* and *V. neocorymbosa* were dominant in the bushland vegetation type. *C.s rotundifolia* was only reported to treat GIN in goats in grasslands and *A. ferox* in bushlands.

## Discussion

Gastrointestinal parasites are a major constraint to goat productivity in many developing countries, leading to high mortality and morbidity [6–8]. The impact of gastrointestinal parasites in goats reared in resource-limited areas is exacerbated by inadequate livestock veterinary care. The extension support delivery system has challenges in resource-limited areas, emanating from a shortage of transport measures, shortage of medication, lack of equipment, and incapacitation, amongst others [18]. Other researchers have reported the poor and failure of extension support systems in other developing countries [19, 20]. Consequently, resource-limited farmers rely on indigenous knowledge to control gastrointestinal nematodes in goats as they possess essential experience acquired through interacting with the environment and their livestock for centuries. Indigenous knowledge contributes to socio-economic growth and sustainable development; therefore, ethnoveterinary plants could be further investigated for anthelmintic activity.

### Household demographics of respondents

The study showed that males headed the majority of households in the bushlands, dominated by the older generation above 50 years. A majority of household heads are members of the Farmer’s Association, where livestock information is usually disseminated through committees. Males interact more with goats at an early age, as they commonly herd and graze livestock. Culturally, women are associated with family care, limiting their involvement in livestock production and health. In addition, women are culturally not permitted to enter kraals, thus it becomes hard for them to practice IK, even though they sometimes possess substantial knowledge [4]. Women need to be involved in goat management as they depend on them for food security and income generation [15].

Formal education involves using conventional methods; therefore, it does not invest in the development of IK theory building and interpretation as the heart of the scientific process, thus influencing the use of IK by those who were informally educated. It was expected that farmers with traditional beliefs use IK more than Christians [15].

### Livestock species kept by farmers

The higher association of the use of IK in goats could be because goats were the earliest domesticated animals reared for food, religious, cultural, and economic reasons from ancient times [21]. The length of the interaction between goats and indigenous people resulted in the evolution of traditional practices in livestock veterinary care. The higher use of IK in smaller herds of livestock could have been influenced by the convenience in sourcing and processing medicinal plants and the labour force involved. Moreover, farmers with smaller herds are perceived as poor [22] because their lower purchasing power limits them from affording conventional medicines.

### Common goat parasites identified by participants

The finding that GIN affected goats from bushlands more than the ones from grasslands could be due to poor nutrition. Such poor nutrition may lead to the susceptibility of goats to diseases due to weaker immune responses, preventing them from coping with the consequences of parasitism and other diseases [23]. Roundworms were of significant concern amongst other gastrointestinal parasites, possibly because of their high fecundity and pathogenicity, causing heavy burdens in pastures, resulting in clinical signs. They cause significant economic impact worldwide [24].

### Methods that farmers use to control gastrointestinal nematodes

The finding that the majority of respondents from both vegetation types used ethnoveterinary medicine more than conventional medicine could be ascribed to the combination of its easy availability, lower cost, practical applicability, effectiveness, one treatment for various diseases, its acceptability in communities, and a claim that it leaves no residues on the meat of treated animals. Research by Sanhokwe et al. [13], Mkwanazi et al. [4] and Ndlela et al. [15] concurs with these findings. Practical training provided by IK holders makes it simple and applicable. The inability to afford anthelmintic drugs could have driven other farmers into using ethnoveterinary medicine [4]. This could justify why some farmers used both conventional and ethnoveterinary medicine, where they could buy drugs when they had money. The parallel use of IK and conventional medicine to control GIN shows the complementarity of these practices. This trend is in consonance with a study by Sanhokwe et al. [13].

### Sources of indigenous knowledge used to control gastrointestinal parasites

Phondani et al*.* [25] reported that indigenous knowledge is orally transferred from one generation to the other and is not fully documented, which could explain why family members are the custodians of IK, especially elderly people from within families and the community at large. Younger generations are unlikely to own livestock due to career advancement, life development, migration to urban areas, and the lack of interest in such practice attributable to the effects of modernisation [26]. They neglect IK as they associate the knowledge with witchcraft and backwardness, making it difficult for the older generation to share knowledge with them [4]. The extraversion and acculturation that characterise modern society need to be addressed.

Farmers share information on animal health care challenges affecting livestock and control measures to curb such ailments during their gatherings, such as farmers' meetings, dipping tanks, auctions. The finding agrees with Luseba and Tshisikhawe [27] that IK was recommended by other farmers, family members and elders. Herbalists and culturalists remain sources of IK since it is more compatible with their personal beliefs and values, however, it was not anticipated that they would not be a major source of IK. It was not surprising that extension services were identified as the least source of IK because they were trained to use conventional methods. Scantlebury et al. [28] indicated that veterinarians, as heads of veterinary services, do not favour the use of IK. The main reason for their failure and other veterinary professionals not to adopt IK could be its lack of scientific validation [25]. Integration of IK into the existing animal health care service could improve communications and contacts between livestock owners, veterinarians and extension support services. This could revive the extension support delivery system and improve service delivery, particularly because IK resources are locally available.

### Indigenous knowledge used by participants to control gastrointestinal parasites

The most frequently mentioned plant families used by farmers to control GIN, *Asphodelaceae, Fabaceae, Vitaceae, Asteraceae* could be due to their vast natural distribution in the area and utilization for multiple diseases, which is a widespread practice in ethnoveterinary medication. Similarly, Williams et al. [29] also identified these families amongst those that are widely used. This might suggest that these families can withstand environmental changes caused by climate change, although several studies have reported a decrease in the number of medicinal plants due to exploitation and environmental degradation [30, 29]. These families are rich in secondary metabolites, such as alkaloids, saponins, flavonoids, tannins, and steroids, enhancing their utilization to treat digestive system problems in livestock and humans [31]. It should be noted that the popularity of these plants does not indicate their effectiveness, which could only be ascertained by efficacy assessment. Such plants could be prioritised for further research to meet farmers’ needs [32].

The frequent use of the *C. quadrangularis* Linn. plant could be due to its natural availability and broad-spectrum. *C. quadrangularis* Linn. is widely used for the treatment of multiple ailments, such as controlling ticks [4], promoting bone fracture and tissue healing [33], treatment of Newcastle disease [34], retained placenta [35] and worm infestation [36]. According to the literature, some popular plants that participants identified have been reported to possess anthelmintic properties amongst other medicinal uses; *A. anthelminthica* Brongn [37], *S. birrea* [38], *T. emetica* [39], *A. ferox* [13], *V. neocorymbosa* [40], and *S. pinnata* [38]. There is scarce literature on the use of *V. xanthophloea*, *A. maculata*, *G. physocarpus*, *C. rotundifolia* and *A. marlothii* as anthelmintics, which shows their unique use in the study area and familiarity through long-term experience. The published literature on *V. xanthophloea* has indicated that it is also used to treat foot and mouth disease [41]. *A. maculata* has been scientifically proven to treat blood scours in calves and enteritis [40].

*G. physocarpus* is used to treat stomach-ache [42]. *C. rotundifolia* is used as a digestive in the food industry [43]. *A. marlothii* uses are not documented, but farmers reported that it has anthelmintic properties like *A. ferox*. The dominance of *A. ferox* and *A. maculata* species used to control gastrointestinal nematode infestation in goats reared under the bushland type corroborate with the findings of Masika et al. [44]. The use of leaves is advantageous because it conserves plants compared to roots, tubers, and the whole plant, which is destructive and unsustainable [45]. The tree bark also followed the same pattern as the leaves. These plant species could be further investigated for their specific activity on different parasite species and for identifying the responsible bioactive compounds, which could help validate IK as a valuable strategy for parasite control. Indigenous knowledge is a potential source of valuable information for sustainable parasite control, and further research should focus on the preservation and analysis of suck knowledge, as well as the scientific evaluation of their effects on animal health.

## Conclusions

The study revealed 12 plant species that farmers use as part of indigenous knowledge to control gastrointestinal nematodes in goats from grassland and bushland vegetation types. Information on the use of IK mostly resonates with older generations; therefore, it could be lost. This, therefore, needs to be documented before they die with the knowledge. The anthelmintic properties of plants claimed by farmers need further scientific validation of their efficacies on parasite infection in vitro and in vivo.

## Supplementary Information

**Additional file 1. **

## Data Availability

The data presented in this study are available on request from the corresponding author. The data are not publicly available due to ethical considerations.
