# Supplementary material for: Characterisation of the indigenous knowledge used for gastrointestinal nematode control in smallholder farming areas of KwaZulu-Natal Province, South Africa
Source: BMC Vet Res. 2022 Feb 21;18:75. doi: 10.1186/s12917-022-03172-0 (PMC8862214; doi:10.1186/s12917-022-03172-0)
Supplement: Supplementary file 1 — Additional file 1. [file 12917_2022_3172_MOESM1_ESM.rtf]

Objective: 
Assessment of farmer perception on the extent of use of indigenous knowledge system to control gastrointestinal nematodes in goats 

Questionnaire number…………………………………..		Village name………………………………
Enumerator name………………..……………………..		Ward number……………………………...
Date……………………………………………………..

SECTION A: Household demographics
A1. Gender of a farmer:	1. M □	2. F □	
A2. Marital status:     1. Married □      2. Single □       3. Divorced □      4. Widowed □
A3. Age:    1. 18-30 □       2. 31-50 □       3. >50 □
A4. Are you residing on the farm?     1. Yes □       2. No □
A5. Highest education level:     1. No formal education □       2. Grade 1-7 □       3. Grade 8-12 □      4. Tertiary □
A6. What is your belief?    1. Traditional □           2. Christian □        3. Both □
A7. What is your employment status?     1. Employed □        2. Unemployed □            3. Self-employed □
A8. What are major sources of income?  1. Crops □    2. Livestock sales □   3. Livestock products □    4. Salary □   
        5. Government grant □   6. Other □, specify ……….
A9. What is your household income?      1. 0-R1000 □         2. R1001=R2000 □       3. Greater than R2000 □
A10. Have you ever received any training on livestock production?            1. Yes □       2. No □
A11. Types of livestock species kept (The last column is for rank levels of the livestock species kept – 1 is for the highest priority)
Livestock species	Number of animals	Rank	
Cattle			
Goats			
Sheep			
Chickens			
Pigs			
Other (specify) …..			


A12. What is the reason for using indigenous knowledge?             
        1. Effectiveness □    2. Availability □   3. Affordability □    4. Quick solution □   5. Works the same as   
        conventional ways □    6. Other □, specify ……….
A13. What is the source of indigenous knowledge?
       1. Oral tradition from parents □       2. Other farmers □    3. Local elders □     4. Herbalists □    5. Culturalists □   6. Extension services □       6. Other □, specify ………………  
A14. Do agricultural institutions promote and support the use of indigenous knowledge to treat animal diseases?    
        1. Yes □       2. No □
A15. How do you transfer indigenous knowledge to other people?
1. Consultation □  2. Selling □   3. Group discussions   4. Children participation □   5. Other□, specify …..  
A16. Which groups within the community use traditional knowledge more?
1. Males □    2. Females □    3. Wealthy □   4. Poor □     5. Young □   6. Elderly □    7. Educated □   8. Non-educated □   9. Other □, specify …… 
A17. Do you see yourself using indigenous knowledge in the future?
1.	Yes □      2. No □
A18. Which method would you recommend for the preservation of indigenous knowledge?
1.	Workshop □   2.  Educate young generation □  3. Include in the syllabus at school □  4. Other, specify □ …

SECTION B: Goat production
B1. Why do you keep goats? (Please tick the first column for the purpose and the second column for ranking -1 is for the highest priority )
Purpose	Tick	Rank	
Meat			
Milk			
Manure			
Skin			
Sales			
Investment			
Traditional ceremonies			
Gifts			
Other (specify)……….			

B2. Are you part of any farmer's association?     1. Yes □       2. No □
B3. Who takes care of goats?    1. Father □       2. Mother □       3. Children □       4. Shepherd □     5. Other □, specify ……….
B4. Who makes decisions about goat management? 
1. Owner □       2. Shepherd □      3. Children □      4. Other □, specify ……….
B5. What goat production system do you use?     
1. Extensive □       2. Semi-intensive □       3. Intensive □        4. Tethering □       5. Integrated livestock/crop system □       6. Other □, specify ……….
B6. Do goats and cattle herds from different households graze together in communal pastures?
	1. Yes □       2. No □ 
B7. Type of vegetation where goats browse?
1. Shrubs □       2. Grass □       3. Tree leaves □       4. Other □, specify ………. 
B8. How has climate change affected the quality of vegetation? 
1.	Dry □      2. Moist/green □    3. No change □
B9. When do you experience feed shortages for goats?
1. Rainy season □   2. Hot-dry season □   3. Cool-dry season □   4. Post-rainy season □   5. All year round □
B10. Do you practice supplementary feeding during periods of feed shortage?     
1. Yes □       2. No □
B11. What supplementary feed do you give to your goats?     
1. Purchased feed □    2. Feed residues □    3. Maize mixed with salt □   4. Mineral licks □   5. Other □, specify ……….
B12. Do you house your goats?     
1. Yes □       2. No □
B13. When do you house your goats?     
1. During the day □        2. Afternoon before sunset □       3. After sunset □       4. Night □       5. Other □, specify ………. 
B14. What form of housing do you have for your goats?
1. Kraal □       2. Stall/Shed □       3. Yard □       4. None □
B15. What are the challenges facing goat production? (Please tick the first column for the challenges and the second column for ranking -1 is for the highest priority)
Challenge	Tick	Rank	
Feed shortage			
Diseases			
Ecto-parasites			
Internal parasites			
Inbreeding			
Theft			
Water scarcity			
Other (specify)……….			

B16. What is the composition of your goat flock?
Goat flock	Male	Female	
Kids			
Weaners			
Does			
Bucks			

B17. How do you breed goats?     
1. Select bucks □       2. Select does □       3. Freely uncontrolled □    4. Tolerance to diseases □
B18. When is the breeding season for goats?
1. Rainy season □	   2. Hot-dry season □   3. Cool-dry season □   4. Post-rainy season □   5. All year round □
B19. What do you look for when selecting bucks? Please tick the first column for the conditions, and the second column for ranking -1 is for the highest priority)
Condition	Tick	Rank	
Scrotal circumference			
Libido			
Body conformation			
Health status			
Scrotal palpation			
Body condition			
Physical injuries			
Other (specify)……….			

B20. How do you select does? Please tick the first column for the conditions, and the second column for ranking -1 is for the highest priority)
Condition	Tick	Rank	
Body condition			
Health status			
Mothering ability			
Ability to produce 3 times in two years			
Other (specify)……….			

B21. How do you manage kids before weaning?     
1. Let them go with mothers to the field □       2. Leave them in the goat house □       3. Leave them in the yard □       4. Other □, specify ……….
B22. When do you wean kids?
1. Rainy season □       2. Hot-dry season □       3. Cool-dry season □       4. Post-rainy season □      
B23. What is your method of weaning?
1. Minimum weight □       2. Age □       3. Feed availability □       4. Health status of doe □      5. Other □, specify……….
B24. Are housed kids provided with water when mothers are being herded?     
1. Yes □       2. No □
B25. How does drying out of water sources affect goat production?
        1. Increased ????        2. Decreased ????
B26. How do production parameters differ between past years and now?
Production parameters	Past years	Present	
	Increased	Decreased	 Increased	Decreased	
Conception rate					
Age at first kidding					
Kidding rate					
Kidding interval					
Kid mortality rate					
Goats mortality rate					

SECTION C: Goat health
C1. What are common disease challenges that you encounter in your flock?  
1. Diarrhoea □   2. Coccidiosis □   3. Heartwater □   4. Orf □   5. Mastitis □   6. Pneumonia □   7. Rift valley □   8. Pulpy kidney □    9. Abortion □    10. Foot abscesses/rot □    11. Anaplasmosis □   12. Lumpy skin □    13. Tick-borne fever □   14. Babesiosis □  15. Anaemia □   16. Bottle jaw □   17. Other □, specify ………. 
C2. What causes kid mortality?
1. Lack of colostrum □       2. No milk produced by lactating does □       3. Predators (Jackals) □          4. Feed shortage □       5. Diseases □   6. Other □ (specify) ………………  
C3. How do you assess health challenges in goats?     
1. Loss of body weight □    2. Breathing difficulties □  3. Not standing/playing □       4. Not eating □       5. Scratching □   6. Diarrhoea □   7. Tearing eyes □  8. Limping □   9. Abdominal swelling □   10. Rash □   11. Coughing/sneezing □    12. Circling □   14. Skin coat rises □   Other □, specify ……..  
C4. What types of parasites are prevalent on this farm? (Please tick the first column for the type of parasites and the second column for ranking -1 is for the highest priority )
Type of parasite	Tick	Rank	
Ticks			
Lice			
Flies			
Mites			
Tapeworm			
Roundworm			
Liver fluke			
Other, specify…			

C5. Are parasite loads affected by housing and grazing land?     
1. Yes □       2. No □ 
C6. Who identifies parasites?     
1. Household head □       2. Shepherd □       3. Other □, specify ……….
C7. What are the different types of gastrointestinal parasites affecting your goats? 
1. Roundworms □   2. Tapeworms □    3. Coccidia □    4. Other □, specify ……….
C8. Do you deworm goats?     
1. Yes □       2. No □
C9. Which method do you use to control gastrointestinal parasites? 
1.	Conventional □             2. Indigenous □              3. Both □

C10. How do you identify a goat that has a problem with gastrointestinal parasites? (The last column for ranking -1 is for the highest priority)
Symptoms	Rainy season	Hot-dry season	Cool-dry season	Post-rainy season	Rank	
Loss of body weight						
Parasites in faeces						
Bottle jaw						
Anaemia						
Post-mortem						
Scours/Diarrhoea						
Stunted growth						
Enlarged abdomen						
Lethargy						
Rough hair coat						
Dry faeces						
Coughing/sneezing						
Fast breathing						
Poor/no appetite						
Other (specify)						

C11. How has the change in rainfall patterns affected the prevalence of gastrointestinal parasites?    
1. Increase □       2. Decrease □       3. No change □
C12. How has the change in temperature patterns affected the prevalence of gastrointestinal parasites?    
1. Increase □       2. Decrease □       3. No change □
C13. What do you use to treat gastrointestinal parasites? 
1. Anthelmintics □       2. Traditional medicine □	       3. Other □ (specify) ……………… 
C14. What are the challenges you have experienced with anthelmintics?       
1. Resistance of gastrointestinal parasites □    2. Expensive □   3. Unavailability □   4. Other □, specify……….
C15. Do you follow the instructions when using anthelmintics?     
1. Yes □       2. No □
C16. What are traditional medicines that you use to control gastrointestinal parasites?
Herb	Plant part (leaves, bark, stem, roots, fruits)	Conditions controlled	Method of preparation (Grind or cook)	Dosage	Recovery period	
Cissus quandrangularis L.						
Vernonia neocorymbosa						
Gomphorcapus physocarpus E.Mey						
Albizia anthelmintica						
Sclerocarya birrea						
Aloe marlothii						
Callilepis laureola						
Aloe maculata						
Clausena anisata						
Clematis brachiata						
Schotia brachypetala						
Agave Americana L.						
Schkuhria pinnata						
Plectranthus madagascariensis						
Othonna natalensis						
Clerodendrum glabrum						
Pittosporum viridiflorum						
Croton pseudopulchellus						
Drimia altissima						
Drimia elata						
Ornithogalum longibracteatum						
Aloe ferox						
Stychnos henningsii						
Pittosporum viridiflorum						
Ipomoea sp.						
Elephantorrhiza elephantina						
Vachellia xanthophloea						
Trichilia emetica						
Euphobia ingens						
Cissus Rotundifolia (Forssk.) Vahl						
Kigelia Africana						
Other (specify)….						
						
